# Reduction Effect of Extra Biochar on PAHs Originating from Corn Stover Pyrolysis

**DOI:** 10.3390/molecules30214238

**Published:** 2025-10-30

**Authors:** Lijie Li, Xiuli Shen, Haibo Meng, Yujun Shen, Jingtao Ding, Hongbin Cong, Mingsong Chen

**Affiliations:** Key Laboratory of Energy Resource Utilization from Agriculture Residue, Ministry of Agriculture and Rural Affairs, Academy of Agricultural Planning and Engineering, Beijing 100125, China; lilijie0101@163.com (L.L.);

**Keywords:** corn stover, extra biochar, polycyclic aromatic hydrocarbons (PAHs), biochar, residual tar

## Abstract

As attention to environmental risks from the PAHs in biochar production increases, developing a low-cost and easy-to-operate optimized pyrolysis process is urgent. The effect of extra biochar was investigated in order to minimize polycyclic aromatic hydrocarbons (PAHs) on biochar and residual tar for the development of a new fixed-bed pyrolysis process. Hence, the effect of extra biochar as a catalyst on the reduction effect on PAHs originating from corn stover pyrolysis was inspected and explored in this study. Pyrolysis was conducted at 500, 600, and 700 °C in a tube furnace reactor with corn stover as the biomass feedstock. Biochar prepared at 500 °C, 600 °C, and 700 °C was used as a catalyst by stacking extra biochar on top of the corn stover raw material. Then, the concentration of PAHs in corn stover biochar and residual tar inside the reactor was examined. The physicochemical characteristics, including morphology, pore structure, and chemical structures of extra biochar, were investigated separately. The results showed that, with stacking extra biochar, the concentrations of PAHs in corn stover biochar (7.15 mg/kg to 1.25 mg/kg) and residual tar (132.23 mg/kg to 101.10 mg/kg) inside the reactor decreased significantly at medium temperatures (500 °C). The concentrations of PAHs in corn stover biochar decreased from 9.14 mg/kg, 10.44 mg/kg to 3.66 mg/kg, 2.7 mg/kg. However, the concentrations of PAHs of residual tar inside the reactor increased significantly at medium temperatures (600 °C, 700 °C). In addition, the reaction mechanism of extra biochar as a catalyst to reduce PAHs in corn stover biochar was established. The results suggest that the measure of adding extra biochar reduced PAHs in resulting biochar effectively, but is not high enough to eliminate PAHs issues in the entire pyrolysis process completely.

## 1. Introduction

Crop straw was one of the most important agricultural resources; the quantity can reach over 865 million tons per year in China. Against the backdrop of “carbon peak” and “carbon neutralization”, the thermal conversion of crop straw into the production of high-quality biochar has been widely implemented. Due to biochar’s ability to improve the physical and chemical properties of soil and promote crop growth, it is widely used in soil remediation [1,2,3,4]. However, before its mass utilization in agricultural adoption, it is necessary to investigate and identify all risks associated with the production process of biochar. Numerous studies have shown that biochar generated from biomass pyrolysis contains polycyclic aromatic hydrocarbons (PAHs), and attention has been paid to the environmental risk from the PAHs potentially generated during biochar production [5,6]. PAHs in biochar were considered to be typical harmful substances due to the pyrolysis process, which was listed as 16 priority-monitored PAHs [7,8]. For this reason, there needs to be concern about the detoxification of the pyrolysis process in the increasing number of designed pyrolysis processes and equipment. In terms of process and equipment design, the production process of the biochar with less PAHs was equally important as energy consumption, production efficiency, and product characteristics, which determine the sustainability of straw pyrolysis technologies. Nevertheless, how to eliminate PAHs generation during the pyrolysis is still a great challenge.

The strategies to minimize the PAH compounds in biochar and pyrolysis systems included regulating the pyrolysis conditions [9], doping heteroatoms (N, B, O, P, and S) [10], and the use of catalysts [11]. The rational design of pyrolysis factors, such as pyrolysis temperature, carrier gas flow, pyrolysis time, and feedstock, was an effective method to inhibit the formation of PAHs during biochar production. In general, the evolution of components produced by biomass pyrolysis was related to pyrolysis temperature [12]; 2–3 rings PAHs are usually formed at low temperature (<500 °C), whereas >4 weight PAHs commonly appear under high temperature (>500 °C) [13]. In addition, heteroatom (e.g., N, B, O, P, and S) doping and metal ion loading have emerged as a promising method of hindering the formation of PAHs during biochar formation. Hung et al. studied that co-doping with N and S suppressed 2-ring PAH formation in pineapple leaf biochar [10,11]. Zhao researched that the pyrolysis of straw loaded with metal ions could suppress the formation of PAHs in biochar [14].

Once biomass pyrolysis began, light gas molecules (H_2_O, acids, and lighter hydrocarbons, etc.) as well as heavy molecular compounds (phenolics, polyaromatic hydrocarbons, etc.) were released, forming pyrolysis vapor in the reactor, and flowed to the reactor outlet. In fact, there was a lack of management of pyrolysis vapor during this process, which can adhere to the solid residue of biomass, the inner wall of the reactor, and other pipelines. Therefore, it is essential to properly handle pyrolysis vapor by primary measures. The behavior of pyrolysis vapors over char at 500~800 °C was investigated in order to maximize tar conversion; the amount of heavy condensable phase decreased [15,16]. Due to the lack of strict separation of pyrolytic vapor fractions from solid residue, the abundance of PAHs in biochar increased [17]. Thus, considering the phase state of the products formed by pyrolysis vapors in actual experiments and the location where their condensation occurred, two types of pyrolytic oil fractions were determined [18]. The bio-oil belongs to the “captured tar” obtained outside the reaction tube, including phenols, aldehydes, and other compounds formed by pyrolysis. Most of the PAHs produced by straw pyrolysis are distributed in bio-oil (94.9~98.9%), and the concentration of PAHs in biochar is relatively small (<5.1%) [19]. It was studied that the distribution of PAHs in birch pyrolysis products found that the proportions of PAHs in the tar collected outside the reaction tank and gas were 62% and 37%, respectively, and biochar contains 0.6% [18]. In addition, “residual tar” refers to the semi-solid tar trapped in the reactor and traps, containing larger aromatic ring systems [20]. Xiong et al. studied this part of tar as “washing tar” from inside the reactor, which is mainly composed of aromatic components [21]. The distribution of PAHs in the residual tar is relatively unexplored.

Recently, the application of catalysts, especially biochar-based catalysts, has emerged as a strategy for enhancing the upgrading of pyrolysis volatile components. Pyo et al. confirmed that the desilicated HZSM-5, with higher mesoporosity and acid site density, reduces the formation of PAH compounds [11]. Wang et al. found small gas molecules from biomass pyrolysis aggregated into the large molecules, and even coke on the biochar-based catalysts [22]. The use of non-in-situ biochar as a catalyst for gas purification and tar reforming has achieved certain results (Gilbert et al., 2009; El-Rub et al., 2008; Pang et al., 2019) [15,23,24]. Li studied the effects of biochar on the generation of gas products, including furans, acids, carbonyl compounds, aromatic aldehydes, CO, CH_4_, etc., during the pyrolysis of sunflower straw [25]. Zhang found that acidic functional groups, such as hydroxyl and carboxyl groups, on the surface of biochar play an important role in tar cracking (Zhang et al., 2019) [26]. Biochar acted roles of a biochar-based catalyst for improving volatile components, and obtaining high value-added products such as biofuel/fuel, biodiesel, chemicals, synthetic gas, hydrogen, aromatic compounds, and functional materials [27,28]. Moreover, biochar has advantages of high specific surface area, porous structure, oxygen functional groups [29], low cost, and sustainability [30]. Therefore, extra biochar could manage and improve volatile components in pyrolysis vapors on large-scale pyrolysis equipment. However, relevant knowledge is relatively lacking.

In this study, the pyrolysis process was implemented by stacking extra biochar specially prepared on top of the corn stover raw material. The concentrations of the 16 US EPA PAHs in the resulting biochars and the residual tar from corn stover pyrolysis were investigated, the correlation between the concentration of PAHs in the resulting biochars and residual tar was analyzed, and the extra biochar was used to trace the development of physicochemical properties by structure and composition characterization methods. The regulatory pathway of extra biochar on PAHs was inferred. The overall objective was to provide a sustainable solution for the demands of current cleaning biochar products and the pyrolysis process, and to develop economically beneficial pyrolysis technology.

## 2. Materials and Methods

### 2.1. Materials

Corn stover (CS) was collected from Harbin city and ground by a grinder (FQ-320, Zhengzhou Yunkai Mechanical Equipment Co., Ltd., Zhengzhou, China), and then it was dried at 105 °C in an oven for 12 h and collected and kept in a drying dish. The extra biochar was corn stover homologous biochar, prepared at different temperatures of 500 °C, 600 °C, and 700 °C, and the PAHs in these biochars were extracted with the Soxhlet extraction method. Therefore, the extra biochar can be considered to contain no PAHs. These biochars as catalysts are named EP-BC-500, EP-BC-600, and EP-BC-700.

### 2.2. Corn Stover Pyrolysis Experiments

**Figure 1:**
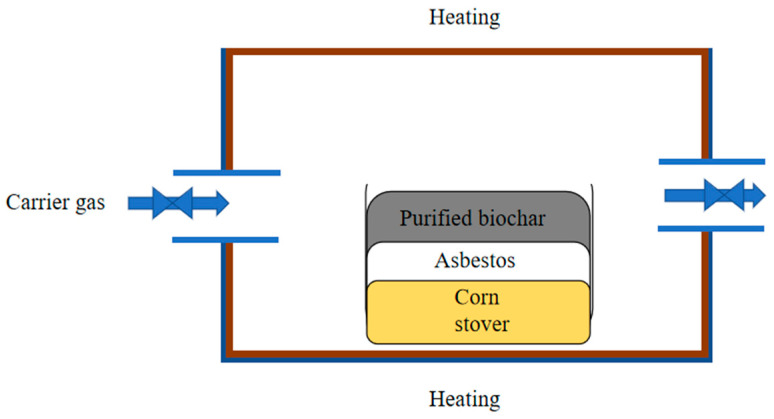
Schematic diagram of pyrolysis test with extra biochar.

The pyrolysis experiment of corn stover was carried out in a quartz tube-equipped fixed-bed reactor (SG-GL1700, Shanghai Institute of Optics and Precision Mechanics, Chinese Academy of Sciences, Shanghai, China) under carrier gas-free conditions. The catalytic zone was located above the pyrolysis zone, at the same location as the reactor, separating zones with quartz wool. The schematic diagram of the pyrolysis test is shown in Figure 1. The inner diameter of the quartz reaction tube is 80 mm and the length is 1000 mm, 20 ± 0.1 g of corn stover samples were placed in a quartz boat in blank groups, and 3 ± 0.1 g extra biochar was stacked in the catalytic zone on the top of 20 ± 0.1 g corn stover raw material in a quartz boat in experimental group. Then, quartz boats were pushed to the central pyrolysis zone of the reaction tube. The reactor was heated at a certain heating rate (20 °C/min) from 25 °C to the preset temperatures (500 °C, 600 °C, and 700 °C). After the reactor reached the preset temperature, it was held at this temperature for 60 min. Once the components were evaporated from the corn stover sample, they formed pyrolysis vapor, and the fresh pyrolysis vapors were passed directly through extra biochar in the catalytic zone. Then, the part of the pyrolysis vapor adhered onto the wall of the quartz tube, forming residual tar, and the portion of pyrolysis vapor outside the reactor was collected as non-condensable gases, a light aqueous phase, and a heavy, viscous tar phase. Tin foil was laid inside the quartz tube to collect residual tar. The resulting biochar and residual tar were a mixture of three parallel experiments. The blank groups were named U500-20, U600-20, and U700-20; the resulting biochars were named CU500-20, CU600-20, and CU700-20. The residual tars were named RTU500-20, RTU600-20, and RTU700-20 in the blank groups. The experimental groups were named ST500-20, ST600-20, and ST700-20, and the resulting biochars were named STRTU500-20, STRTU600-20, STRTU700-20, and the residual tars were named STCU500-20, STCU600-20, and STCU700-20 in the experimental groups. After the pyrolysis reactions, the extra biochars as catalysts on the quartz wool were collected and named EP-BC-500A, EP-BC-600A, and EP-BC-700A.

### 2.3. Analytical Methods

Sixteen PAHs were identified and quantified by gas chromatography–mass spectrometry (GC 7890B and MS 5977B, Agilent, Hercules, CA, USA) with an equivalent column (30 m × 250 μm × 0.25 μm HP–5MS UI, Agilent, Hewlett, CA, USA). All determination experiments were performed in triplicate. Sixteen kinds of PAHs can be divided into categories: (1) Low molecular weight PAHs (L-PAH) containing 2–3 rings, including naphthalene, acenaphthylene, acenaphthene, fluorene phenanthrene, and anthracene; (2) Medium molecular weight 4-ring PAHs (M-PAH), such as fluoranthene, pyrene, benz(a)anthracene, and chrysene; and (3) High molecular weight PAHs (H-PAH) with 5–6 rings, including benzo(b)fluoranthene, benzo(k)fluoranthene, benzo(a)pyrene, dibenz(a,h)anthracene, benzo(g,h,i)perylene, and indeno(1,2,3-c,d)pyrene. The abbreviations of 16 US EPA PAHs could be founded in Table S1, Supplementary Materials. 

The morphology of biochar was observed by a scanning electron microscope (SEM, JSM-IT200, JEOL Ltd., Tokyo, Japan). The extra corn stover biochars were treated with gold spray. The structural features of biochar were characterized by Raman spectroscopy (InVia, Renishaw plc, London, UK) with a laser at a wavelength of 785 nm, and the calculation method of feature parameters followed [12]. The surface area and pore analysis were conducted by a fully automated specific surface area and porosity analyzer (Autosorb-IQ-MP, Conta, Somerset, NJ, USA), with high-purity nitrogen (99.999%) as the adsorbent and a testing temperature of 77.35 K. The distribution characteristics of biochar were tested by using an X-ray photoelectron spectroscopy analyzer (K-Alpha+, Thermo Fisher Scientific, Waltham, MA, USA). The vacuum in the analysis room was approximately 5 × 10^−9^ mbar, and the X-ray source was a monochromatic AlKa source (Mono AlKa) with an energy of 1486.6 eV, a voltage of 15 KV, and a beam current of 15 mA. The scanning mode of the analyzer was CAE.

### 2.4. TEQ

The Toxic Equivalency Factor (TEF) of PAHs in biochar is mainly used to evaluate the toxicity of biochar. The toxicity of each PAH monomer is related to its molecular weight, and high molecular weight PAHs typically exhibit higher toxicity. In order to accurately evaluate the toxicity of PAHs in biochar, TEQ was calculated based on the toxicity equivalence factor (TEF) and the concentration of each PAH monomer according to Equation (1).

(1) $$TEQ=\sum_{1}^{n}\left(C{PAH}_{i}\times TEF_{i}\right)$$

In the formula, *CPAH_i_* is the concentration of PAHs, mg/kg; *TEF*_i_ is the toxic equivalence factor of each PAH monomer.

## 3. Results and Discussion

### 3.1. PAHs in Biochar

**Figure 2:**
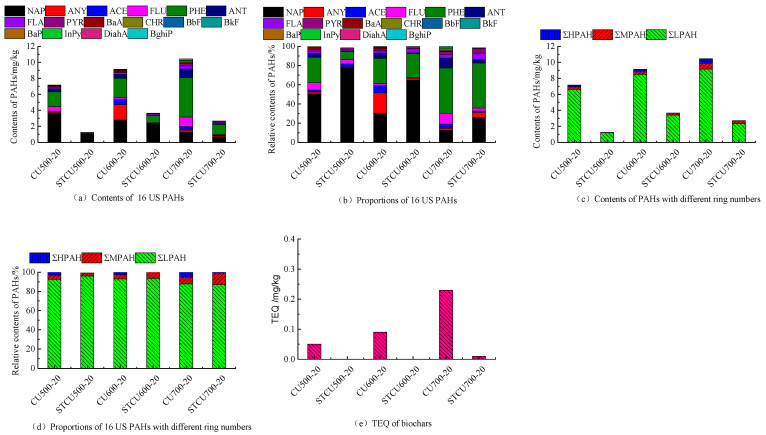
The concentration, fraction, and TEQ of PAHs in corn stover biochar at different pyrolysis temperatures.

Figure 2a presents a temperature increase from 500 to 700 °C, resulting in a rise in PAHs concentration from 7.15 mg/kg to 10.44 mg/kg in corn stover biochar generated when pyrolysis was performed. The highest concentration in CU600-20 and CU500-20 is NAP, while in CU700-20 it is PHE (Figure 2b). The trend of NAP descended from 3.60 mg/kg to 1.29 mg/kg with pyrolysis temperatures. On the contrary, the trend of PHE increased from 1.88 mg/kg to 4.92 mg/kg. It can be inferred that NAP undergoes thermal cracking, due to its conversion as intermediates from medium to HPAHs [10,31]. ANY and ACE undergo a trend of first increasing and then decreasing, indicating that the consumption pathway of 3-ring PAHs transfers a greater number of rings [32]. The PHE monomer is generated by stripping alkyl groups from alkylated forms of PHE [33]. The abundance of LPAH (NAP, ANY, ACE, FLU, PHE, ANT) in total PAHs can reach 87.88~93.02%, following the order: CU600-20 > CU500-20 > CU700-20 (Figure 2d). The concentrations of MPAH (FLA, PYR, BaA, CHR) in CU500-20, CU600-20, and CU700-20 range from 0.51 mg/kg to 0.76 mg/kg (Figure 2c). Low concentration of HPAH (0.07 mg/kg~0.51 mg/kg) has only been observed in CU600-20 (BaP, BghiP) and CU700-20 (BbF, BkF, BaP, DiahA, BghiP InPy) (Figure 2c). The proportions of different ring-number PAHs depended on the pyrolysis temperatures [34].

With extra biochar, PAHs concentrations in corn stover biochar newly generated rose from 1.25 mg/kg to 3.66 mg/kg with a temperature increase from 500 °C to 600 °C (Figure 2a), and then decreased to 2.7 mg/kg upon further temperature increase to 700 °C (Figure 2a). Compared with the blank groups without extra biochar, the concentration of PAHs in biochars from the experimental groups with extra biochar decreased up to 59.94%~82.45% (Figure 2b). It was speculated that extra biochar protected newly generated biochar in the period of pyrolysis reaction, and the PAHs formation mechanism changed during the pyrolysis process due to extra biochar. These PAHs may be mainly formed through the solid-state PAHs formation mechanism [35]. The impact on PAHs formation of the secondary reaction of vapors was weakened. For PAH categories, with extra biochar, the distribution of different ring-number PAHs mainly focused on LPAH (NAP, ANY, ACE, FLU, PHE, ANT) and MPAH (FLA, PYR) in STCU500-20, STCU600-20, and HPAH almost disappeared except for STCU700-20 (Figure 2c,d). LPAH showed a slight increase with the pyrolysis temperature ranging from 500 °C to 600 °C, and a decrease with the temperature up to 700 °C. MPAH increased with the pyrolysis temperature from 500 °C to 600 °C. The reason may be that LPAH was consumed to form larger molecules. At high temperature, the decrease in the concentration of free PAHs is related to small aromatic rings fused into nonextractable large aromatic systems [33].

The toxicity equivalent of biochar produced by corn stover pyrolysis was shown in Figure 2e. Results revealed that the toxicity equivalent of biochars from the blank group without extra biochar increased from 0.05 mg/kg to 0.23 mg/kg as the pyrolysis temperature increased. The TEQ value produced by the pyrolysis of corn stover with extra biochar at different temperatures varied from 0 to 0.01 mg/kg.

The extra biochar layer has an impact on the content and types of PAHs in the newly generated biochar. The extra biochar had adsorption capacity due to its porous structure and various oxygenated functional groups, and various chemical reactions of volatile components occurred on the extra biochar layer. In addition, the extra biochar provided an obstruction when vapors recondensed and adhered to the newly generated biochar. The TPAH content of the biochar newly generated goes below the lowest threshold according to the International Biochar Initiative (6 mg/kg) and European Biochar Certificate (4 mg/kg) [10]. The pyrolysis process of adding extra biochar reduces harmful substances in newly generated biochar, and environmental risks are reduced.

### 3.2. PAHs in Residual Tar

**Figure 3:**
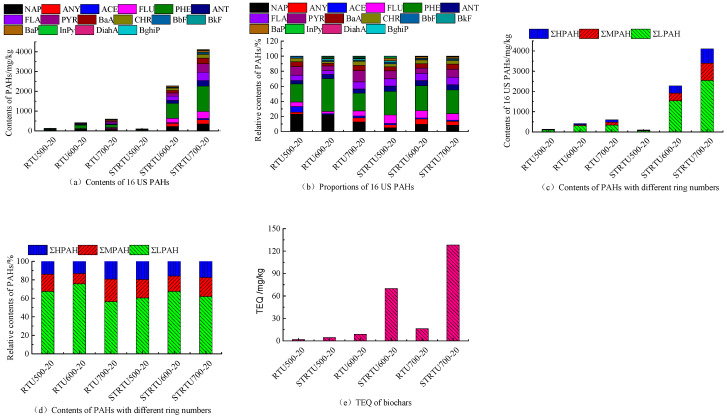
Concentration, fraction, and TEQ of PAHs in residual tar.

The concentration of PAHs in the residual tar produced by the corn stover pyrolysis in the blank groups without extra biochar is shown in Figure 3a. The overall concentration of 16 US PAHs and the concentration of each PAH monomer in the residual tar of RTU500-20, RTU600-20, and RTU700-20 significantly increased. Notably, the overall concentration of 16 US PAHs in the residual tar soared sharply from 101.10 mg/kg to 413.51 mg/kg, indicating that 500 °C~600 °C is a critical temperature for PAHs formation during the pyrolysis. When the pyrolysis temperature rose to 700 °C, the concentration of 16 US PAHs in the residual tar increased up to 600.40 mg/kg. The concentration of LPAHs, MPAHs, and HPAHs in the residual tar increased from 89.18 mg/kg, 24.58 mg/kg, and 18.47 mg/kg to 312.99 mg/kg, 46.18 mg/kg, and 54.34 mg/kg, respectively, at 500 °C, 600 °C, and 700 °C (Figure 3c). The reason is related to the increase in the release amount of PAHs and alkyl compounds of PAHs during corn stover pyrolysis at the temperature range [12]. The alkyl compounds of PAHs, such as alkyl-naphthalene, alkyl-phenanthrene, cracked and generated free radicals, and further formed thermodynamically more stable PAH (e.g., NAP, PHE) [33]. The process of pyrolytic PAH production at high temperature was complex, which involved numerous reactions. Cyclopentadiene from phenols through Diels-Alder reaction and C-H β-scission precipitated PAHs formation [36]. Hydrocarbons and monocyclic benzene series formed PAHs by the hydrogen abstraction and the phenyl addition pathway [37,38]. However, there is a trend of increasing first and then decreasing in proportion of LPAHs, MPAHs, and HPAHs with temperatures increasing (Figure 3d), and the concentration of naphthalene was highest at RTU600-20 (Figure 3a). This result indicated that the 2-ring NAP was also the precursor for higher PAH formation in gas-phase reactions. The toxicity equivalent of PAHs in the residual tar increased from 1.74 mg/kg to 16.29 mg/kg with temperatures increasing (Figure 3e). The high toxicity of the residual tar has never received enough recognition, affecting the cleanliness of the entire pyrolysis system.

The concentration of PAHs in the residual tar slightly decreased, and the concentration of each PAH monomer at 500 °C with extra biochar, the proportions of LPAHs, and HPAHs increased from 60.29%, 10.14% to 67.44%, 13.97%. However, the proportions of MPAHs decreased from 29.57% to 18.59%. At 600 °C and 700 °C, compared with the blank groups without extra biochar, the overall concentration of 16 US PAHs and the concentration of each PAH monomer in the residual tar significantly increased. In addition to the dissociation and reaggregation of aromatic compounds in pyrolysis vapors, the promotional effect of the extra biochar on the free radicals reaction was also an important reason [21]. It may be that oxygen-containing functional groups provide a carrier for electron transfer for free radical reactions. At higher temperatures, the concentration of 16 US PAHs in the residual tar increased up to 2280.00 mg/kg and 4110.00 mg/kg, respectively. Extremely high values of 16 US PAHs concentration in the residual tar indicated that the strategy for adding biochar is still incomplete.

### 3.3. Correlation Analysis of PAHs Concentrations Between Biochar and Residual Tar

Their relationship in terms of PAHs concentrations between the two productions, biochar and residual tar, in the pyrolysis reactor, could provide information to explain the source of PAHs in biochar. The correlation analysis between the concentration of PAHs in biochar and residual tar is shown in Table 1. It can be seen that, in the blank groups without extra biochar (500 °C, 600 °C, and 700 °C), significant correlation (*p* < 0.01) between the concentration of PAHs in biochar and residual tar indicates that the source of PAHs in biochar may be related to residual tar generated from the secondary reaction and condensation of volatile components of vapors. Thus, the concentration of residual tar is a parameter to assess the impact of biochar on PAHs [34]. With extra biochar in the experimental groups (500 °C, 600 °C), there was no correlation in the concentration of PAHs between biochar and residual tar, indicating that there is an inhibitory effect of additional biochar on PAHs in newly generated biochar. However, a significant correlation (*p* < 0.01) was found between the concentration of PAHs in biochar and residual tar at 700 °C, whether additional biochar is added or not. This phenomenon may be related to the massive formation of PAHs at high temperatures [36]. Reducing the opportunity for residual tar formation is a way to avoid biochar contamination by PAHs, and these results can provide guidance on the issue of residual tar in pyrolysis reactors.

### 3.4. Biochar Characteristics

#### 3.4.1. SEM

**Figure 4:**
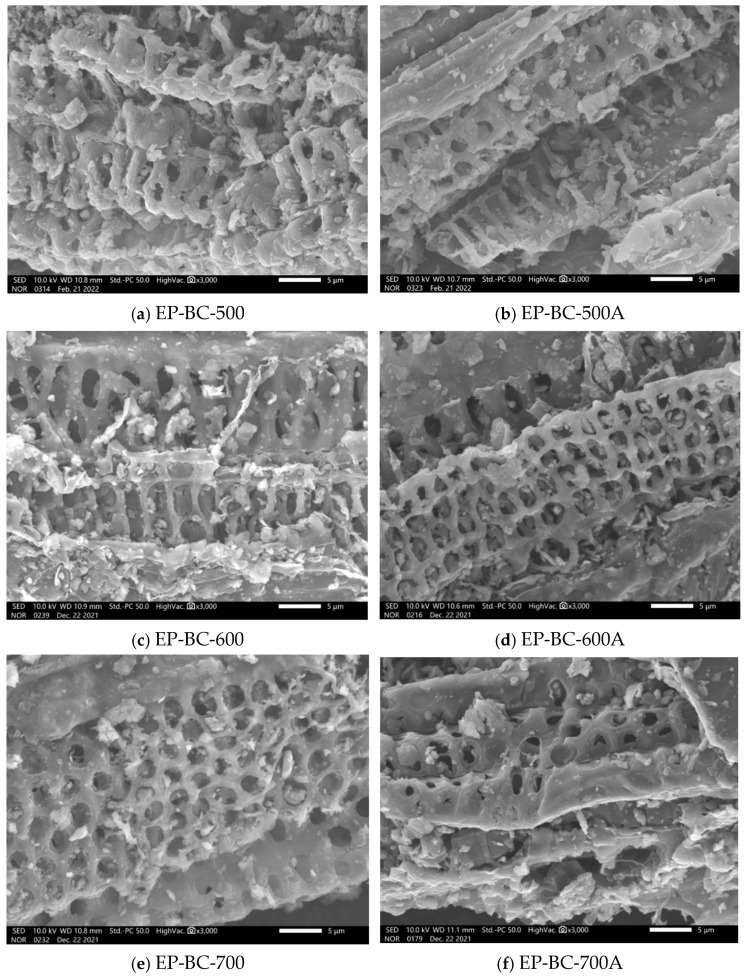
SEM images before and after purification of biochar as catalyst.

The SEM images of extra biochar produced by corn stover pyrolysis at 500 °C, 600 °C, and 700 °C are shown in Figure 4. It can be seen that the extra biochar has a rough surface and rich pore structure in its original state. The surface of the extra biochar has an open pore structure from the inside out, which allows the volatile components generated from the corn stover pyrolysis not only to react on the surface of the extra biochar but also to reach the interior of the extra biochar through such pores. After the pyrolysis reaction of corn stover, the pore structure of the three kinds of extra biochar is still clearly visible. It is indicated that the extra biochar remains loose and porous during the pyrolysis process, making the volatile components fully in contact with reaction sites [22].

#### 3.4.2. BET

The specific surface area and pore size analysis of extra biochar are shown in Table 2. It is shown that the BET specific surface area of extra biochar has a certain value, indicating a limited adsorption capacity for the components volatilized from corn stover pyrolysis due to its relatively poor porosity [22]. The surface of extra biochar prepared at 500 °C is rough, and the specific surface area remains almost unchanged after reaction. The micropores (<2 nm) slightly increase, which may be due to the volatile components connecting and filling the microstructure of the extra biochar when vapors pass through the extra biochar layer. The biochar prepared at 600 °C did not form a rough surface due to the high degree of graphitization of the carbon skeleton. The polymerization of volatile components appeared in the pores, resulting in a decrease in specific surface area and a slight decrease in mesoporous (2–50 nm) and microporous area. The pores of extra biochar prepared at 700 °C collapsed. However, the mesoporous pores increased after repairing and reconstructing with tar components. From the microscopic morphology analysis, it can be seen that the volatile components of biochar undergo a process of transformation from connection and aggregation to reconstruction at different temperature stages.

#### 3.4.3. Raman Analysis

**Figure 5:**
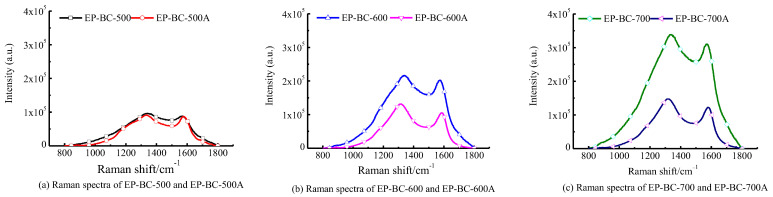
Raman spectrum of extra biochars.

The total Raman spectra intensity represented the total amount of oxygen-containing functional groups. The Raman spectra of extra biochar at the temperatures of 500 °C, 600 °C, and 700 °C are shown in Figure 5. In Figure 5, it can be seen that the total spectral area of extra biochar prepared at 500 °C, 600 °C, and 700 °C has slightly decreased, indicating a decrease in the content of oxygen-containing functional groups on the surface of biochar.

The Raman spectrum was deconvoluted by the 10 peaks method, and characteristic peak intensity ratios are shown in Table 3. The ratios I_D_/I_G_, I_S_/I_G_, and I_D_/I_(GR + VL + VR)_ represent the four states of carbon element in biochar, and explain the evolution of aromatic structure of biochar. It is shown that the ratios of the biochar catalysts undergo a significant change and show the same trend after pyrolysis as the catalysts. That is, for ID/IG and IS/IG, they all decrease to varying degrees, indicating that the degree of graphitization increases, the content of branched chain structures decreases, and the proportion of large ring structures with ≥6 rings increases. This means that with extra biochar, the degree of graphitization increased, the content of branched chain structures, including sp^3^C-C,C-H, and ether bonds C-O decreases, and these results are confirmed by the XPS analysis. As a matter of fact, it was considered that the ether bonds C-O and sp^2^C C=C were part of the linkage bonds that constituted carbon atoms in biochar [39]. The volatile components generated from pyrolysis undergo a free radical recombination reaction with the branched chains on the extra biochar layer when passing through it, forming a graphite structure [20], and growing and renewing biochar [21]. ID/I_(GR + VL + VR)_ increased to varying degrees. This indicates that after purifying biochar, the proportion of large ring structures with ≥6 rings increases, and the aromatic hydrocarbon structures with 3–5 rings decrease and are transformed into large ring structures with ≥6 rings [40]. This phenomenon indicates that after the adsorption of tar by extra biochar, the components or free radicals in the tar are assembled onto the extra biochar-bone, which may be related to the capture effect of the branched chains, thereby reducing the number of branched chains and transforming the small ring structure (3–5) into the large ring structure (greater than 6) through polymerization. Combining Raman spectra analysis of extra biochar catalysts, the extra biochar layer above the corn stover reduced HPAHs in the biochar produced from the pyrolysis of corn stover. The reason may be that there are aromatic components containing PAHs in the volatile components that combine with the extra biochar branch chains when passing through the extra biochar layer, transforming them into aromatic components with a larger number of rings, which constitutes the carbon skeleton structure for purifying biochar. It can be inferred that the upgrading of the aromatic ring system is due to the active sites and volatile components from pyrolysis dehydrogenation and deoxygenation polymerization on biochar [22].

#### 3.4.4. XPS

**Figure 6:**
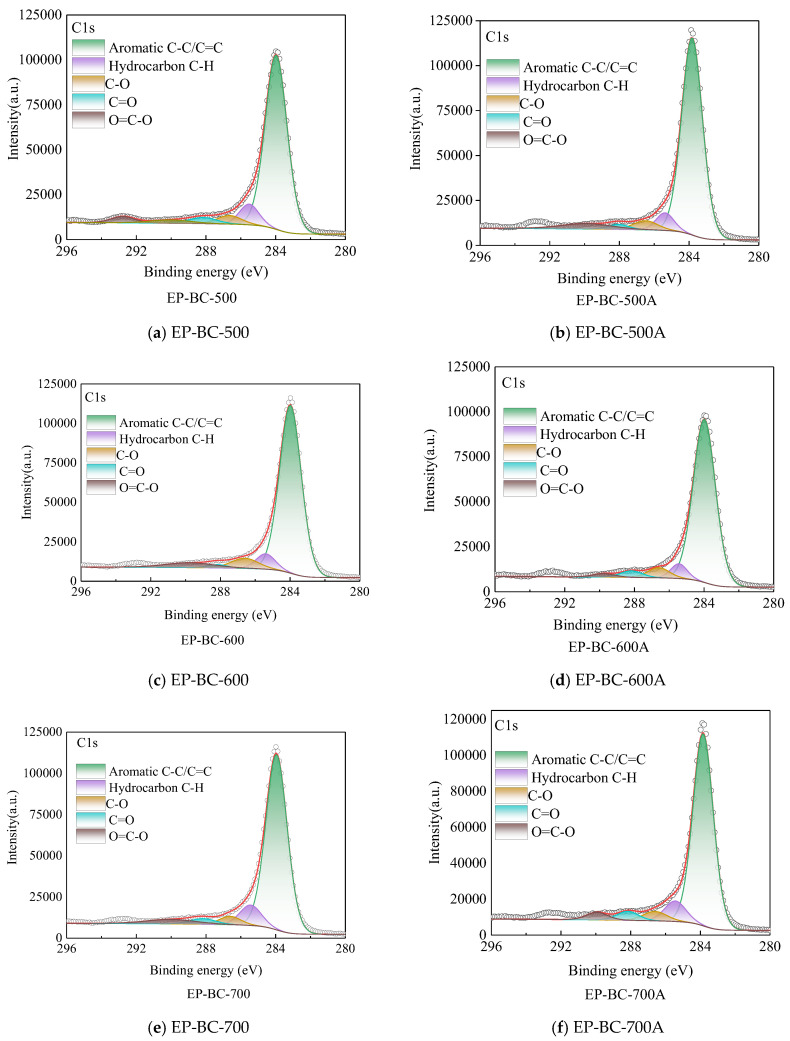
The C1s spectra of extra biochar as catalyst before and after the reaction (the circle symbol represents the fitted curve).

The presence of carbon elements on the surface of biochar catalysts was further investigated and analyzed. Fine spectral analysis was conducted on the C1s orbitals of biochar catalysts, and the fine spectrum of biochar revealed the changes in its surface functional groups before and after the reaction. The C1s in the XPS analysis of extra biochar are shown in Figure 6. There was a significant difference in the C1s spectrum of extra biochar as a catalyst in XPS analysis before and after pyrolysis. Figure 6 shows an asymmetric C1s peak, indicating the presence of complex carbon forms in biochar. The C1s peak were deconvoluted into peaks of different categories corresponding to sp^2^ C (284.46 ± 0.3 eV), sp^3^ C (285.15 ± 0.3 eV), and oxygen-containing groups such as C-O (286.23 ± 0.2 eV), C=O (287.49 ± 0.3 eV), and O=C-O (288.66 ± 0.4 eV) in extra biochar catalyst. The sp^2^ C reflected the aromatic structure in biochar catalysts, while sp^3^ C generally belongs to aliphatic chain structures. After using 500 °C, 600 °C, and 700 °C extra biochar catalysts during corn stover pyrolysis, the surface carbon element content increased, up to 3.48%, 1.46%, and 7.43%, respectively. This indicates that the volatile components generated by corn stover pyrolysis undergo transformation on the surface of the extra biochar and are assembled on the extra biochar matrix, consistent with the results of Raman analysis.

**Figure 7:**
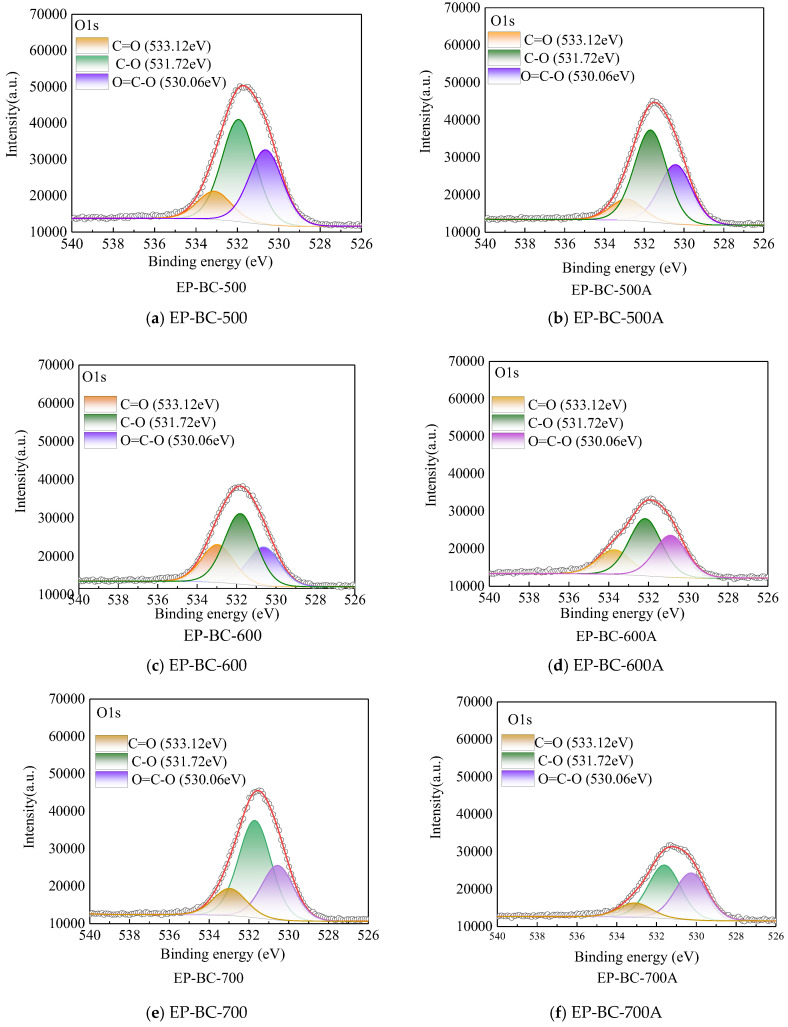
The O1s spectra of extra biochar as catalyst before and after the reaction (the circle symbol represents the fitted curve).

The O1s spectra of the three kinds of biochars are shown in Figure 7. It can be observed that oxygen atoms exist in the form of C (O), such as carbonyl C=O, phenols\alcohols\ethers C-O, and carboxyl O=C-O structures. Nevertheless, when reacted as a catalyst during corn stover pyrolysis, although the peak shape of O changed, the C (O) peaks distribution showed no evident change. Compared with that of EP-BC-500, EP-BC-600, and EP-BC-700, the proportions of oxygen atoms in EP-BC-500A, EP-BC-600A, and EP-BC-700A showed a descending trend, up to 3.74%, 1.49%, and 7.31%, respectively. The peaks of carbonyl C=O, phenols\alcohols\ethers C-O, and carboxyl O=C-O on the three kinds of extra biochar catalyst shrank to different degrees, generally. And for the extra biochar catalyst at temperatures of 500 °C, 600 °C, and 700 °C, carboxyl O=C-O, carbonyl C=O, and ether bonds C-O, respectively, tended to be active sites on the surface, which is conducive to the cracking of volatile components during corn stover pyrolysis.

In the process of using extra biochar as a catalyst to adsorb volatile components from corn stover pyrolysis, the C-C and C-H bonds in the extra biochar increase, while the C (O) structure decreases. This is mainly due to the reforming mechanism of the extra biochar, which gradually converts the volatile components from corn stover pyrolysis into a part of the extra biochar. The carbon-oxygen bonding structure C (O) may be the specific site connecting the volatile components from pyrolysis.

### 3.5. Analysis of Pathways for Reducing PAHs in Biochar

When pyrolysis was performed without extra biochar, the volatile components may undergo polymerization reactions on the newly generated biochar, forming PAHs through secondary reactions of vapors [31]. In the experimental group with extra biochar, the volatile components generated by corn stover decomposition are adsorbed by the extra biochar, polymerization reaction occurs, and part of the carbon elements in the vapors are transferred to the extra biochar, which can be confirmed by Raman and XPS analysis results. This leads to a decrease in the migration of carbon elements into the newly generated biochar in the gas phase. Therefore, this may be the reason for the reduction of PAHs in the newly generated biochar in the experimental group with extra biochar.

Briefly, from the change concentrations of PAHs in newly generated biochar in the experimental group with extra biochar, it can be seen that the formation of PAHs in biochar is mainly due to the secondary reaction of vapors (up to 59.94~82.45%), and there are relatively few PAHs formed through the solid-phase mechanism [35]. Therefore, weakening secondary reactions of vapors is the main direction of minimizing PAHs formation in biochar. To this end, to place acceptors for the migration of carbon elements in vapors would be a promising measure.

**Figure 8:**
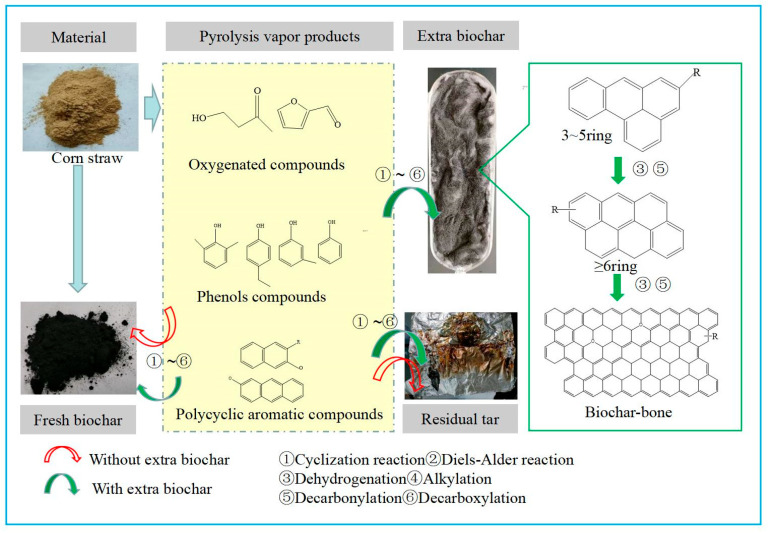
Pyrolysis vapor products aggregating into the large molecules of carbon-bone on extra biochar.

At high temperature, the temperature at which PAH growth is most active [34], it is observed that the extra biochar plays a limited role, indicating that the concentration of the PAHs in residual tar is dependent primarily on the tubular reactor. Some studies have proved that there are two main sources of PAHs in biochar, and their formation mechanisms generally include solid phase and gas phase [34]. Compared with the solid phase mechanism, it is necessary the gas phase mechanism is almost rarely discussed, especially [35], because the contribution of the gas phase reaction mechanism should not be determined. Here we consider the PAHs formation of gas phase reaction mechanism through the cracking of phenols from corn stover pyrolysis. In volatile components of vapors, phenols are one of the important precursors of PAHs formation in the gas phase for lignocellulosic biomass [35,36,41]. Phenols play an important role in the formation of PAH synthesis pathways [14,31]. For slow pyrolysis reactions, phenols are released before 600 °C in the slow pyrolysis reaction of corn stover [12]. Phenol monomers generated from the lignin cracking might be difficult to recombine as the large aromatic ring systems (>3 rings) [42]. However, the active sites on the surface of biochar promote the further cleavage of phenols. There was an adsorption mechanism based on acid–base interaction and hydrogen binding between phenol and the functional groups in biochar [43]. When the pyrolysis temperature reaches 600 °C and 700 °C, biochar undergoes further aromatization, reducing the release of substances, mainly aromatic hydrocarbons without heteroatoms. Thus, the hydrogen binding adsorption has weakened between aromatic hydrocarbons and the C(O) of biochar catalyst, resulting in an adsorption mechanism between phenol and the functional groups in biochar and reducing polymerization of phenol on biochar. PAHs formation of gas phase reaction mechanism through cracking of phenols from corn stover pyrolysis contributes to the residual tar at high temperatures (>600 °C), continuing to aggregate into larger rings (Figure 8).

## 4. Conclusions

In light of the dual imperatives of safety and environmental sustainability in biochar production, this study systematically investigated the catalytic effects of exogenous biochar on PAH reduction during corn stover pyrolysis. Through controlled experiments employing a layered biochar-corn stover configuration across varying pyrolysis temperatures (500–700 °C), three key findings emerge. The introduction of exogenous biochar induced distinct temperature-mediated effects. At 500 °C, significant reductions in PAH concentrations were observed both in produced biochar (82.52% decrease) and residual tar (23.54% decrease). Conversely, at elevated temperatures (600–700 °C), while biochar-bound PAHs decreased by 59.96–74.14%, residual tar PAH content increased by 4.51–5.85 times, suggesting thermal regime-dependent catalytic pathways.

The exogenous biochar functioned as an effective catalytic matrix through the provision of active sites, biochar surface functional groups analysis, and carbon skeleton integration. This work highlights two critical considerations for pyrolysis system design, one of which is the necessity for targeted reactor modifications to address tar accumulation. The economic viability of biochar-mediated process optimization requires less energy input than the conventional pyrolysis process. These findings advance the fundamental understanding of secondary catalytic pyrolysis while providing actionable strategies for sustainable biomass valorization. Future research should focus on reactor-level tar mitigation techniques and life-cycle assessment of the proposed methodology.

## Figures and Tables

**Table 1:** Correlation analysis of PAHs in biochar and residual tar. ** *p* ≤ 0.01.

Sample	U500-20	ST500-20	U600-20	ST600-20	U700-20	ST700-20
Correlation coefficient	0.862894	0.059431	0.751019	0.452046	0.798589	0.885022
Statistical significance	**		**		**	**

**Table 2:** Analysis of specific surface area and pore size of extra biochar.

Sample	BET Surface Area (m^2^/g)			Pore Volume (cm^3^/g)		Pore Size (nm)		
	Total	Mesopore	Macroporous	Mesoporous	Macroporous	Mesopore	Macroporous	Average Value
EP-BC-500	3.30	2.32	3.206	0.013	0.013	1.410	3.799	15.67
EP-BC-500A	3.29	2.54	3.30	0.012	0.012	4.75	4.91	14.25
EP-BC-600	3.08	2.20	3.55	0.013	0.013	4.75	3.80	17.06
EP-BC-600A	2.92	2.05	2.94	0.011	0.012	1.41	3.85	15.78
EP-BC-700	2.03	1.56	2.46	0.011	0.010	4.75	4.92	20.37
EP-BC-700A	2.45	1.84	3.30	0.012	0.012	4.54	4.93	19.57

**Table 3:** Raman parameters extra biochar as a catalyst before and after the reaction.

Extra Biochar	I_D_/I_G_	I_S_/I_G_	I_D_/I_(GR+VL+VR)_
EP-BC-500	2.08	1.13	0.32
EP-BC-500A	1.40	0.63	0.38
EP-BC-600	2.77	1.73	0.37
EP-BC-600A	1.98	0.71	0.63
EP-BC-700	2.34	1.25	0.44
EP-BC-700A	2.02	0.65	0.66

## Data Availability

The data presented in this study are available on request from the corresponding author.

## Supplementary Materials

The following supporting information can be downloaded at: https://www.mdpi.com/article/10.3390/molecules30214238/s1. Table S1. The abbreviations of 16 US EPA PAHs.

## Abbreviations

The following abbreviations are used in this manuscript:

PAHs	polycyclic aromatic hydrocarbons
FTIR	Fourier transform infrared
BET	Balanced-Emitter Transistor
SEM	scanning electron microscope
XPS	X-ray photoelectron spectroscopy
TEF	Toxic Equivalency Factor
TEQ	toxic equivalency
