# Supplementary material for: Reduction Effect of Extra Biochar on PAHs Originating from Corn Stover Pyrolysis
Source: Molecules. 2025 Oct 30;30(21):4238. doi: 10.3390/molecules30214238 (PMC12609004; doi:10.3390/molecules30214238)
Supplement: Supplementary file 1 [file molecules-30-04238-s001.zip › molecules-3481473-supplementary.pdf]

**Table S1.** The abbreviations of 16 US EPA PAHs

| **Compounds** | **Abbreviation** | **Unit** | **Detection limit** |
|---|---|---|---|
| Naphthalene | NAP | mg/kg | 0.09 |
| Acenaphthylene | ANY | mg/kg | 0.09 |
| Acenaphthene | ACE | mg/kg | 0.12 |
| Fluorene | FLU | mg/kg | 0.08 |
| Phenanthrene | PHE | mg/kg | 0.10 |
| Anthracene | ANT | mg/kg | 0.12 |
| Fluoranthene | FLA | mg/kg | 0.12 |
| Pyrene | PYR | mg/kg | 0.12 |
| Benz(a)anthracene | BaA | mg/kg | 0.12 |
| Chrysene | CHR | mg/kg | 0.14 |
| Benzo(b)fluoranthene | BbF | mg/kg | 0.17 |
| Benzo(k)fluoranthene | BkF | mg/kg | 0.11 |
| Benzo(a)pyrene | BaP | mg/kg | 0.17 |
| Dibenz(a，h)anthracene | DiahA/DBA | mg/kg | 0.13 |
| Benzo(g，h，i)perylene | BghiP | mg/kg | 0.13 |
| Indeno(1，2，3-c，d)pyrene | InPy/IcdP | mg/kg | 0.12 |
